# Environmental sensitivity in young adolescents: The identification of sensitivity groups in a Polish sample

**DOI:** 10.1371/journal.pone.0271571

**Published:** 2022-07-21

**Authors:** Monika Baryła-Matejczuk, Grzegorz Kata, Wiesław Poleszak

**Affiliations:** Institute of Psychology and Human Sciences, University of Economics and Innovation in Lublin, Lublin, Poland; Wroclaw University of Economics and Business: Uniwersytet Ekonomiczny we Wroclawiu, POLAND

## Abstract

The research described herein is based on the assumptions of the sensory processing sensitivity concept and the meta framework for the concept of environmental sensitivity. The adopted theoretical framework shows that individuals differ in their sensitivity to the environment, with some being more sensitive than others. From the evolutionary perspective, it has also been assumed that sensory processing sensitivity follows a normal distribution in the population, with a minority being exceptionally or highly sensitive to environmental stimuli. We explored data from a sample of 928 young adolescents in two studies. The tool used to evaluate their sensitivity was the Highly Sensitive Child Scale, which in studies 1 and 2 had a three-factor structure. Latent class analysis was used for the interpretation of the data of the studied groups. The obtained results indicate the existence of three groups which differ significantly from each other according to the HSC result. Based on the obtained results, it may be assumed that young adolescents are divided into three groups characterized by different sensitivities and their percentage distribution is not in agreement with the research conducted to date. The acquired information has both a theoretical value and a practical applicability, prompting reflection about the different aspects of the study, such as cultural differences, changes related to the development stage and the characteristics of the evaluation tool itself. From the perspective of possible applications, the obtained results may provide important information (1) to decision-makers who plan support or intervention programs at various levels of prevention, (2) for practitioners to provide them with the means with which to consider sensitivity as an important factor in coping with difficulties through diversified and adequate support (3) that is broadly applicable in the face of an environmental crisis (pandemic, the changing structure of class groups which is related to the number of refugees).

## Theoretical foundations

Environmental sensitivity is defined as the ability to perceive, register and then process external stimuli [1]. Environmental sensitivity is also defined as one of the most basic individual characteristics and may be observed not only in humans but also in most animal species [2]. This feature allows the individual to be able to perceive, evaluate and react to various environmental conditions, both of a physical and psychosocial nature. According to the assumptions of the concept of environmental sensitivity (e.g., [1–4]) both humans and animals are programmed to perceive, process, react and adapt to specific environmental conditions—both positive and negative [5]. The concept of environmental sensitivity is referred to as an umbrella term [6]. It is a concept that is superior to other theories used to explain individual differences in the ability to register and process environmental stimuli. It includes the Differential Susceptibility concept [7, 8], the concept of Sensory Processing Sensitivity [9, 10] as well as Biological Sensitivity to Context [11, 12]. A common feature of the above-mentioned concepts is the view that both people and animals show diverse levels of sensitivity in response to supportive as well as to difficult, adverse environments, with some individuals being significantly more sensitive than others to the negative effects of adversity, stress and related difficulties of a psychological nature, but also "disproportionately susceptible to the beneficial effects of supportive and enriching experiences (or just the absence of adversity)" ([8] p. 885). This means that the explanation for this phenomenon had been sought by conducting research concerning reactivity to the environment, both in terms of physiological mechanisms, reactivity to stress and phenotypic manifestations (e.g., [11, 13]).

The concept of Sensory Processing Sensitivity (SPS) assumes that temperamental traits play a key role in these differences. SPS has been presented as a manifestation of environmental sensitivity [1, 6, 10] and people characterized by a high level of this trait are referred to as highly sensitive [9].

## Literature review

This review is divided into two groups of research studies. The first group is concerned with research on the psychometric properties of tools that have been developed to date in order to measure the sensory processing sensitivity and environmental sensitivity. A more accurate description of the subject is given by considering the factor structure of the tools. The second group includes studies that deal with attempts to determine the distribution of the SPS trait in the population. From an evolutionary perspective, it has been assumed that this trait follows a normal distribution in the population, with a minority being particularly or highly sensitive to environmental stimuli [8, 14, 15].

The first measurement factor for the evaluation of sensory processing sensitivity, which was developed as a result of research conducted in this field, was the Highly Sensitive Person (HSPS) scale [10]. Research conducted by Aron & Aron [10] contributed to the formulation of a definition of SPS, which refers to a wider construct for processing various types of stimuli/information coming from the environment. It was not assumed that SPS is a straightforward, one-dimensional sensitivity to stimuli and the factor analyses of the results obtained using the HSP-scale suggested the existence of a unitary sensitivity factor. Also, in other studies, it was indicated that a one-factor scale provided the best explanation of the obtained results [16, 17]. Alternatively, one of the most frequently cited studies concerning the psychometric properties of the HSP scale indicate its non-uniformity [18]. Data analyses of the study conducted on a sample of students showed favourable compliance rates for both the original Aron and Aron’s model and their three-factor model, with the chi-square fit for the three-factor model being significantly better. The identified factors were designated as Low Sensory Threshold (LST), Ease of Excitation (EOE) and Aesthetic Sensitivity (AES). These terms are widely accepted in many studies and reviews, and the individuals factors have been the subject of numerous studies (i.a., [6, 19]). The division into three separate factors is also the most representative aspect of the shortened Norwegian version of the scale [19], the Polish version [20], and also of one of the German versions [21]. However, it should be emphasized that some of the test items in the cited studies were removed with reference to the original version of the Aron scale (i.a., [18, 19]), and some other ones were added [21]. In turn, other adaptations of the tool and the corresponding research studies which used these tools indicate, inter alia, the two-factor structure of the tool (i.a., [22–25]), or four-factor structure [26, 27], or else five-factor structure [28]. The HSP scale is mainly used to examine adults, although the abbreviated version has been used in studies involving adolescents [29]. In order to examine children and adolescents, the Highly Sensitive Child (HSC) Scale was developed. The basis for its layout was the HSP scale [2] and the tool itself was also used to evaluate the sensitivity of preschool children [30]. A factor analysis of the scale showed that the HSC scale has sufficient internal consistency and favourable psychometric properties for independent samples [2]. In addition, research conducted using the HSC scale confirmed that children who achieved high scores on this scale are more sensitive and respond positively to psychological intervention [31]. Furthermore, the longitudinal multi-informant study indicates that SPS may correlate with individual differences in susceptibility to both positive and negative quality parenting [30]. Moreover, it is widely known from the studies conducted to date that although the trait itself is not a disorder, the coexistence of high sensitivity with psychological and psychiatric problems has been demonstrated among highly sensitive people growing up in unfavourable conditions (cf., [16, 32, 46, 47]).

In the subject literature, we find some reports that environmental sensitivity has a normal distribution in the population, and that about 20% of people are highly reactive to the environment (e.g., [11]). The results of several studies indicate the percentage of highly sensitive people in the population (i.a., [2, 11, 14, 15, 33]). In order to test or confirm the hypothesis about the presence of identifiable groups characterized by different levels of sensitivity, with the identification being based on sensory processing sensitivity or environmental sensitivity, latent class analysis (LCA) was used in the research conducted to date. Initially, an assumption was made that highly sensitive people are undoubtedly present in the population and an *orchid* analogy was used in this case, which describes people who perform exceptionally well in favourable conditions and exceptionally badly in unfavourable, aversive and poorly stimulating environments. Moreover, there are also people with low sensitivity to whom the *dandelion* analogy was applied, it refers to those who are more mentally resilient and to whom environmental conditions are not of significant importance to their development (i.a., [11]). In the research by Pluess et al. [2] and also Lionetti et al. [14] the presence of a third group was also demonstrated—moderately sensitive people (this subgroup was found among adults as well as children) who were compared to *tulips*.

The intensity of SPS may be important for physical, emotional and social development [13], but also for the quality of life [34] or, as it may be assumed, in professional development, (ie in making decisions, in management and cooperation) (cf., [35, 36]). Research conducted on a sample of 901 healthy adults who were examined using a shortened 12-item version of the HSP scale (which reflects a bifactor model with a general sensitivity factor) showed that people characterized by high sensitivity were present at a rate of 29.19% in the total sample (in the subsamples 26.58% - 30.00%), moderately sensitive people at the rate of 40.29% (in the subsamples 42.15% - 44.67%) and the low sensitivity group was present at the rate of 30.52% (in the subsamples 25.33% - 31.27%) [14].

The studies by Pluess et al. [2] were conducted in Great Britain using the HSC scale, and indicate that 34.90–34.98% of adolescents belong to the low sensitivity group, 41.04–46.90% to the moderately sensitive group, and 21.20–23.97% to the highly sensitive group. However, the authors assume that specific features related to cultural background are present in the sample. Since the evolutionary approach would assume the absence of intercultural differences as a relatively invariant trait in different populations, other researchers [15] attempted to confirm the assumptions on a sample of German teenagers. The research was conducted on a sample of teenagers attending schools in Germany (a sample of 749 teenagers) and was carried out using a shortened 10-item version of the Highly Sensitive Person Scale, which in research by Tillmann, El Matany, & Duttweiler [29] displays a structure characterized by two correlated factors (Sensitive Openness to Stimuli and Overexcitability/Negative Effect from Overstimulation). The results of these studies confirmed the existence of three groups distinguished by their sensitivity with their corresponding mean HSP scores being significantly different from each other. According to the results obtained by researchers [15, p.9] “134 (17.90%) of the adolescents belonged to the low sensitivity group, 413 (55.10%) were placed in the medium sensitivity group, while 202 (27.00%) formed part of the high sensitivity group”. Differences may be noted between the sizes of the groups in the above-cited studies, e.g. in the sample of German teenagers, the low-sensitivity group was smaller than in the British group, and the moderate- and high-sensitivity groups were larger.

In summary, we also wish to draw attention to the importance of measuring temperamental traits, and in particular conducting further research in the area of environmental sensitivity. In the analysis of psychological features in general, one should take into account both the conditions of the environment in which a person develops, and the cultural context of Poland [37] and of Europe as a whole [35, 36, 38, 39]. The trait of SPS is associated with the occurrence of special benefits, especially if the person develops in favourable conditions (cf., [6, 30, 31]) alternatively difficulties may occur if the person develops in particularly difficult conditions [50, 51]. Creating a supportive environment for highly sensitive children and youth may be likened to creating an organizational culture, especially from the perspective of creating a more pro-environmental culture (cf., [37]). In this approach, it is crucial to develop assumptions that support the appropriate adaptation (external and internal integration) in difficult life situations, and because the mechanisms are effective they can be transferred to other community members [37, 40].

Taking into account the results of the studies conducted to date and the characteristics of the functioning of highly sensitive people, three main objectives of the research presented herein were established. The first objective is concerned with the analysis of the psychometric properties of the tool used for the high sensitivity evaluation of both children and adolescents. An assumption was made that the studies conducted using a tool with validated psychometric properties would be a source of important scientific information and an important element of the implementation work (supporting highly sensitive children). The second objective of the study is to verify the number and size of the groups of young people characterized by different levels of environmental sensitivity. Given that high sensitivity may be both a risk factor and a protective factor–knowing which group may be affected by a particular environmental impact is crucial for both practitioners (when designing adequate support) and researchers analysing the cultural differences in the distribution of the trait in different populations. The research conducted to date will be extended with the results of an analysis of a Polish sample of young adolescents.

## Materials and methods

### Participants and procedure

The data described in this article are the result of studies of two separate groups of adolescents carried out one year apart. A factor analysis was performed in the first and second study. A replication of the exploratory factor analysis on a similar data set was used as a solution to confirm the factor structure and ensure that the same definitions of the subscales will be observed in the new samples [40].

The study group consisted of primary school students who participated in the project of the University of Economics and Innovation which focused on identifying the students’ abilities and the factors that determine them. Participation in the project, and thus in the tests using the HSC scale was voluntary, the participants were recruited at school level. Several schools gave consent for their students to participate. Ethical approval was obtained from the University of Economics and Innovation. The research was carried out as part of the standard ‘mass testing’ during the implementation of the project. The study was conducted according to the guidelines of the Declaration of Helsinki, and approved by the Institutional Review Board of University of Economics and Innovation in Lublin (No. 4/05/2020, 13.05.2020). The participants, apart from consenting to study in the project (which took place at the school level), expressed their verbal consent to participate in the study (prior consent of the students’ parents was obtained, which was documented in the research protocol). The verbal consent was given in the presence of the teacher participating in the lesson during which the study took place. Verbal consent was sought at the individual level, during the instruction period, the students were informed about purpose of the study, procedures involved, risks and benefits of participation, opportunity to withdraw, confidentiality of the data, and contact persons in case individuals needed further. The data were analyzed anonymously. A group of 439 students participated in the first study. Their mean age was M = 11.17 years (range 10–14 years; SD = 0.764). A year later, 489 people with an average age of M = 10.95 (range 10–14 years; SD = 0.780) participated in the second round of the study.

In the first study, the percentage of girls and boys was highly similar (49.7% to 50.3%). Study 2 was dominated by females (57.3%) (Table 1). The participants were primary school students. Entire class groups were examined, among which most were in 5^th^ year (49% in the first study and 51.5% in the second). Most of the schools were located in cities. Rural schools accounted for about 20% of the total in both measurements.

**Table 1:** Descriptive statistics for studied group.

		Study 1		Study 2	
		N	%	N	%
Gender	Female	218	49.7	280	57.3
	Male	221	50.3	209	42.7
Year (Grade)	4	58	13.2	44	9
	5	215	49	252	51.5
	6	166	37.8	193	39.5
Place of study	Urban area	332	75.6	391	80
	Rural area	107	24.4	98	20

The data of all of the students surveyed were used in the analysis. There were no gaps or errors obstructing the filling in of the questionnaires. A factor analysis was performed on the data from the first and second measurements, to check the convergence of the results. A latent class analysis was performed on a combined group of 928 students.

### Measurements

Environmental sensitivity was evaluated using the HSC scale [2]. A Polish version of the scale was developed by the customization of the 12-item version of the scale which was translated into Polish using the back-translation procedure. Initially, HSC was independently translated by two qualified psychologists with psychometric experience. Then the accuracy of the translation was verified and the scale was re-translated into English. The final version was translated into Polish and edited by a team of psychologists with knowledge of English, so that it fully corresponded to the Polish cultural context. The translation procedure was carried out using the Protocol for the Adaptation of Questionnaires developed by the author of this tool. The participants of the study answered the questions following the Likert scale ranging from 1 to 7 points (with the scale ranging from “1 = strongly disagree”, to “7 = strongly agree”). The mean score across all items was computed in order to create the total score with higher scores reflecting a higher degree of sensitivity. Cronbach’s alpha was high with α = 0.72 [95% CI 0.68, 0.75]. Examples of items from the questionnaire include the following: “Loud noises make me feel uncomfortable”, “I don’t like watching TV programmes that have a lot of violence in them”, “I don’t like it when things change in my life”, “I love nice tastes”.

### Data analysis

The factor structure was analysed using Principal Component Analysis (PCA). The Varimax rotation and Kaiser normalization were used. The number of components was selected based on eigenvalues greater than 1 and on scree plot interpretation. In order to identify the groups with different intensities of sensory processing sensitivity, a series of Latent Class Analyses (LCA) was perfomed. LCA is a method of analysing a set of variables (sample items; indicators or manifest variables), as a result, subgroups are distinguished among the respondents (latent classes). Each of the identified groups consists of subjects similar to each other in terms of the analysed variables. In other words, the result of the method is such a division into groups that maximizes the differences between them and minimizes the differences between people belonging to a given group. LCA is performed several times, starting with a solution without divisions (number of classes is one) and then checking the options with two or more categories [41, 42]. The selection of the most optimal solution is based on the values of the coefficients, which constitute the matching criteria. This article is based on the following fit parameters: Bayesian information criterion (BIC), sample-size adjusted Bayesian information criterion (ABIC), consistent Akaike information criterion (CAIC). The rule is to choose the solution with the lowest values of the above-mentioned coefficients. Comparative analyses show that, among the above parameters, the most reliable indicators are ABIC and AIC (after [43]). Both values take into account the size of the tested sample. After selecting the number of groups, the value of the Entropy coefficient was also checked. It provides information about whether the selected solution correctly classifies the respondents into separate subgroups. The expected value is equal to or greater than 0.80. In order to summarize, when choosing the number of classes, preference is given to those solutions where most of the calculated quantitative criteria achieve the lowest value. Theoretical premises and diagnostic utility are also important (cf., [42]).

LCA is a method comparable to other grouping methods, such as cluster analysis. Its main advantage is that it provides results that are more relevant to the data at hand. This is mainly due to the fact that the decision concerning the number of groups is based on matching factors, which are the formal rationale for choosing one solution over the other. Moreover, it is a technique relating to the probability of people belonging to specific groups and not to the so-called "cut-off points". The latter may cause people similar to each other in terms of the analysed variables to end up in two different groups (cf., [42, 44]). In the present study, the validity of a division into 1 to 6 groups was checked. This range was selected in order to compare the results with those obtained by other researchers: Pluess et al. [2] and Tillmann et al. [15]. The analyses were performed using the SPSS version 25 program and the R environment with the poLCA package [45].

## Results

### Factor analysis

The factor analysis included the respondents’ answers to all 12 items. In the first step, the sampling adequacy measures of the first and second study were checked. The Kasier-Meyer-Olkin ratio reached a value of 0.752 for the first set of data and 0.676 for the second set. In relation to individual items, the value of the coefficient exceeded the limit of 0.5 (cf., [46]). The obtained KMO values were considered satisfactory.

The choice of the number of factors was guided by the Kaiser criterion (initial eigenvalues greater than 1) and the shape of the scree plot. On this basis, it was decided to adopt a solution based on three factors. This solution explained a total of 47.78% of the total variance of the data collected in the first study and 43.61% in the second study (Table 2). Obtaining the same structure in two studies justifies the formation of three dimensions describing sensory sensitivity and allows for a generalization of the results for the population of adolescents aged 10–14 years. Details concerning a comparison of the factor loadings from the first and second group may be found below in the description.

**Table 2:** Sum of squares of charges (study 1 and study 2).

Component		Sums of squares of loadings after extraction			Sums of squares of loadings after rotation		
		Total	% of Variance	% Cumulative	Total	% of Variance	% Cumulative
Study 1	1	3.114	25.951	25.951	2.271	18.928	18.928
	2	1.510	12.580	38.531	1.767	14.724	33.653
	3	1.110	9.249	47.780	1.695	14.127	47.780
Study 2	1	2.496	20.796	20.796	1.961	16.339	16.339
	2	1.462	12.181	32.978	1.700	14.165	30.504
	3	1.276	10.634	43.612	1.573	13.108	43.612

Table 3 shows the factor loadings for all 12 items. They were calculated after the orthogonal rotation was applied. As a result of the analysis of the correspondence of test items to factors, the following dimensions were defined: Factor 1- Ease of Excitation (EOE), Factor 2—Aesthetic Sensitivity (AES), Factor 3- Low Sensory Threshold (LST). The dimensions were defined on the basis of the content of the test items and their names were taken from the original version of the tool ([2] cf., [18]). The scale is one-dimensional, the overall result is of key importance, and the obtained dimensions provide a supplementary description of the respondents.

**Table 3:** Factor loadings of the test items in studies 1 and 2.

Number and content of the question		Component		
		1 (EOE)	2 (AES)	3 (LST)
Study 1	4. I get nervous when I have to do a lot in little time	0.748		
	6. I am annoyed when people try to get me to do too many things at once	0.735		
	12. When someone observes me, I get nervous. This makes me perform worse than normal	0.625		
	8. I find it unpleasant to have a lot going on at once	0.545		
	9. I don’t like it when things change in my life	0.446		
	10. I love nice tastes		0.748	
	3. I love nice smells		0.691	
	5. Some music can make me really happy		0.686	
	1. I notice when small things have changed in my environment		0.318	
	11. I don’t like loud noises			0.805
	2. Loud noises make me feel uncomfortable			0.686
	7. I don’t like watching TV programmes that have a lot of violence in them			0.609
Study 2	6. I am annoyed when people try to get me to do too many things at once	0.802		
	4. I get nervous when I have to do a lot in little time	0.800		
	12. When someone observes me, I get nervous. This makes me perform worse than normal	0.535		
	8. I find it unpleasant to have a lot going on at once	0.448		
	9. I don’t like it when things change in my life	0.249		
	3. I love nice smells		0.751	
	10. I love nice tastes		0.747	
	5. Some music can make me really happy		0.532	
	1. I notice when small things have changed in my environment		0.290	
	11. I don’t like loud noises			0.786
	2. Loud noises make me feel uncomfortable			0.737
	7. I don’t like watching TV programmes that have a lot of violence in them			0.548

When interpreting the values of the factor loadings for individual test items, the large size of the samples in studies 1 and 2 was taken into account (cf., [41]). For this reason, values such as 0.249 for item no. 9 and 0.290 for item no. 1 were considered sufficient.

The same factor structure was obtained in the first and second study. The highest loadings for each item were observed for the same factors. The differences between the values of the factor loadings were also checked. For each of the twelve questions, the difference is less than 0.2, which allows one to assume that the identified dimensions and their components provide a reliable description of the general population of adolescents aged 10–14 years (after: [31]). Cronbach’s Alpha Reliability Analysis for the overall scale score returned a value of 0.723 for the first study and 0.634 for the second study. It is a satisfactory result with regard to the diagnostic and research utility of the scale.

### Latent class analyses

The analyses described in this part of the article were used to identify groups of tested individuals who are similar to each other in terms of their sensitivity of sensory processing. The number of such groups, their size and the differences between them were determined. The results of the Polish sample are presented in Table 4 below. The data represents the validation of the options for an absence of division and divisions into one to six groups. The factors listed in the table are used to select the most optimal solution. The calculations included the responses of the testees to 12 test items from both stages of the study (N = 928).

**Table 4:** Results of the LCA (n = 928).

Number of classes	log-likelihood	BIC	aBIC	cAIC	Entropy
1	-19214.71	38921.39	38692.73	38993.39	-
2	-18610.85	38212.49	37751.98	38357.49	0.81
3	-18272.83	38035.26	37342.92	38253.26	0.80
4	-18106.11	38200.62	37276.44	38491.62	0.80
5	-18012.30	38511.82	37355.79	38875.82	0.81
6	-17917.91	38821.86	37433.99	39258.86	0.76

**Fig 1:**
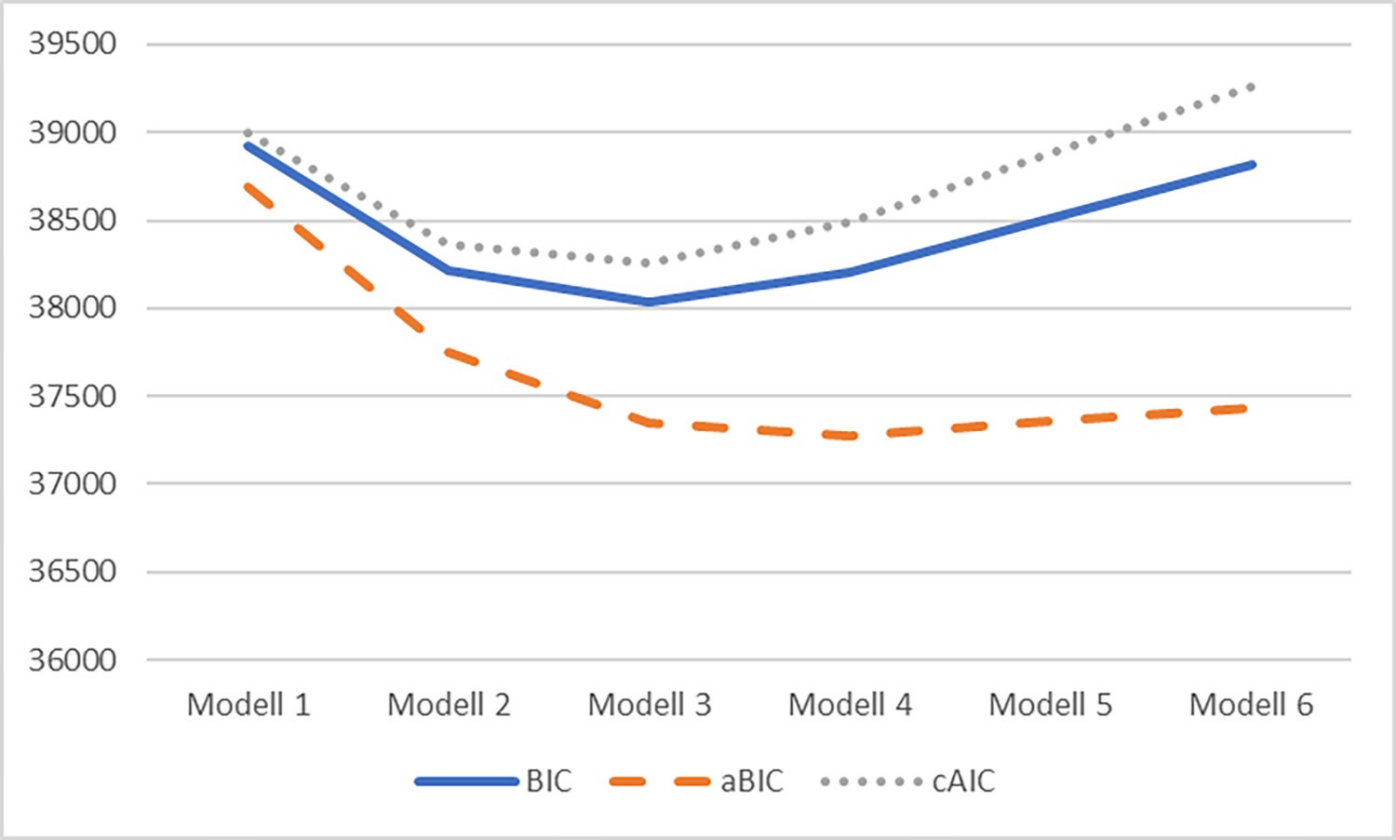
Information criteria of LCA.

The fit indices in Table 4 prove that a solution based on a division into three groups is the most viable option. Two of the three factors (BIC, cAIC) reach the lowest values for this solution and the entropy coefficient reaches a threshold value of 0.80. The choice of three-class solutions is also justified by the shape of the Fig 1, in which an obvious decrease is noted in the third modelled curve (cf., [42]).

The three groups of adolescents distinguished using LCA are characterized by different levels of sensory processing sensitivity. The first group, representing 37.7% of the adolescent respondents, includes highly sensitive individuals. The second group included adolescents with an medium trait level (20.5% of the studied population). The last and the largest group (41.8%) is represented by students with the lowest total score on the scale, who are not highly sensitive. The Table 5 below shows the group averages and the result of their comparison.

**Table 5:** Descriptive statistics and a comparison between groups of students with different levels of sensory processing sensitivity.

Group	M	SD	F	p
Lowest sensitivity	47.80	6.749	354.955	0.000
Medium sensitivity	58.41	12.497		
High sensitivity	63.06	5.609		

The groups differ significantly in the intensity of sensory processing sensitivity (F (2.925) = 354.955, p <0.05). Sheffe’s posthoc tests also show a variability in the overall HSC score.

Results that are worth noting were obtained for adolescents belonging to the group with an average overall intensity of sensitivity. A high standard deviation (SD = 12.497) is related to the presence of young people with low, high and medium results in this group. These groups are, however, not separate in terms of the level of the overall score, but they differ in the specificity of the responses and thus in their functioning within the individual features that constitute the sensitivity of sensory processing. Additional information is provided in Table 6 below, which describes the results using subscales.

**Table 6:** Descriptive statistics for HSP-scale dimensions.

	EOE Ease of Excitation subscale		AES Aesthetic Sensitivity subscale		LST Low Sensory Threshold	
Sensitivity group	M	SD	M	SD	M	SD
Low sensitive group	17.78	4.158	20.84	4.091	9.18	3.289
Medium sensitive group	24.15	7.520	22.86	4.601	11.41	5.970
Highly sensitive group	25.73	3.672	23.23	2.857	14.09	3.345

Young people with an medium level of sensitivity are characterized by the greatest differentiation in the EOE subscale (SD = 7.520), while the group of highly sensitive people is characterized by the least differentiation of the result of this subscale. Similarly, the highly sensitive group is the least diverse in the case of AES, and in turn, the differentiation of the results of the other two groups is comparable. In the case of the LST subscale, we may observe a comparable differentiation of extreme groups and the highest degree of differentiation is present in the medium sensitivity group.

## Discussion

The obtained results are only partially consistent with the results of other authors who studied the issue of selecting groups with different sensitivity levels. The obtained results confirm the presence of three groups differing in their sensitivity in the population of adolescents, which is in agreement with research conducted by Pluess, et al. [2] and Tillmann et al. [15]. In both of the above-mentioned studies, the same method of analysis was used as in the present article. The percentages of individual groups described by Pluess et al. [2] ranged from 20–35% for the highly sensitive group, 41–47% for the moderately sensitive group and 25–35% for the low sensitivity group. While the group of highly sensitive people may be regarded as comparable, in the research on young adolescents conducted in Poland, the size of the moderately sensitive group is smaller, and the low-sensitivity group is larger than in the study above. In turn, Tillmann et al. [15] showed the following division: 14.29% for the low-sensitivity group, 53.27% for the moderately sensitive group and 32.44% for the group with the highest score, so in this case, the difference in the percentage contribution between the low and average sensitivity groups is even greater. The results of the moderately sensitive group are particularly interesting. The total intensity of the feature measured using the HSC scale allows for their assignment to a moderately sensitive group (using the previous analogy—to tulips), however, a high standard deviation may indicate the presence of young people with low, high and average scores in various areas of sensitivity (in various questions) in this group. The intensity of the trait significantly differentiates the three groups, however, the average of the overall score is taken into account. Therefore, it may be assumed that the groups, apart from being divisible in terms of the overall score, also differ in the specificity of their responses and thus functioning within the features that correspond to the sensory processing sensitivity. The results obtained indicate that the intensification of certain behaviours related to sensitivity (such as anxiety or feeling unwell when facing multiple tasks in a short time or at the same time) may be characteristic not only for highly sensitive people but also for the developmental period associated with emotional lability and often indicates a change in the threshold of responsiveness (cf., [47, 48]).

A question arises about the differences in the sizes of the groups with different sensitivity levels. They may be related to cultural differences, to changes associated with the stage of development as well as to the properties of the psychometric tool itself. It is also worth noting that all of these three areas are interconnected. We may conclude from the results of the research that, as previously assumed [6], highly sensitive people constitute the minority in society. The commonly used comparison with orchids gives the impression that this group is particularly sensitive to the effects of the environment. There is an important practical implication for the sensitivity of the groups detected. In the conducted research, it is important to pay attention to the practical consequences of our findings. Orchids, due to their increased sensitivity to positive stimuli, may be more sensitive to psychological interventions, school-oriented interventions to support mental resilience as well as anti-bullying interventions [31, 49, 50]. This is important information for decision-makers designing support programmes or intervention programmes at different levels of prevention. Moreover, the knowledge that almost 1/3 of the respondents are highly sensitive according to the results described herein, may lead one to question, in some way, the suitability of widespread educational methods or if all of the students should be provided with the same kind of support. The results are especially important having knowledge about the possible difficulties of highly sensitive children raised in an unfavorable environment, the coexistence of sensitivity with psychiatric problems such as depression or alexithymia or have a negative impact on quality of life (cf., [16, 34, 51, 52]).

## Conclusions

The results above provide answers to the two research questions formulated in this article. The factor structure of the Polish version of the HSC scale for children aged 10–14 has a three-factor structure. Separated subscales, based on the content of items with the highest factor loadings, were defined as Ease of Excitation (EOE), Aesthetic Sensitivity (AES), and Low Sensory Threshold (LST). The three-factor solution is supported by studies using both the HSC scale [29] and the HSP scale [18, 21]. The main purpose of using the scale is to evaluate the sensory processing sensitivity and the overall result provides such information. The subscales are of a complementary nature.

The criteria for the selection of the most optimal solution for the number of groups with different sensitivities was obtained as a result of Latent Class Analysis, this favoured division into three groups of young people. Each of them had a significantly different overall HSC score. Students with the highest level of the examined feature constituted 37.7% of the total population. The medium sensitivity group constitutes 21% of the tested population, and people with the lowest sensitivity made up the remaining 41.8% of the population.

## Limitations and future directions

The sensitivity groups identified as a result of this study are to be reproduced in independent samples. It is particularly interesting to reproduce the analyses in older groups of adolescents, as well as to perform in-depth analyses in the group of moderately sensitive Polish teenagers. These analyses may allow researchers to further explore the specific traits of the distinguished sensitivity groups. In addition, they enable practitioners to use the HSC scale to evaluate sensitivity at an individual level. Such an identification of the trait seems to be crucial for the provision of adequate support to highly sensitive children and adolescents. Identification may be a key step towards more personalized intervention or prevention programmes that take into account the provision of adequate conditions for the development of people with different sensitivity levels (while considering the benefits and challenges of having a different intensity of the trait). Another direction of research which is important in terms of the value of the application are intercultural studies concerning the subject of the HSC scale which seems to have the potential for adaptation to many cultures, therefore comparisons may be of particular value in the evolutionary approach to the subject (cf., [11, 14, 15, 33]). In future research, it is worth exploring both the influence of the cultural aspects as well as some developmental and age-related aspects that may be the reason for the differences in the percentages of the selected sensitivity groups.
